# Increased Expression of Circulating microRNA 101-3p in Type 1 Diabetes Patients: New Insights Into miRNA-Regulated Pathophysiological Pathways for Type 1 Diabetes

**DOI:** 10.3389/fimmu.2019.01637

**Published:** 2019-07-23

**Authors:** Aritania S. Santos, Edecio Cunha Neto, Rosa T. Fukui, Ludmila R. P. Ferreira, Maria Elizabeth R. Silva

**Affiliations:** ^1^Laboratório de Carboidratos e Radioimunoensios - LIM/18, Hospital das Clinicas HCFMUSP, Faculdade de Medicina, Universidade de São Paulo, São Paulo, Brazil; ^2^Heart Institute (InCor) and Division of Clinical Immunology and Allergy - LIM60, University of São Paulo School of Medicine, São Paulo, Brazil; ^3^Institute for Investigation in Immunology, National Institutes of Science and Technology (iii-INCT), São Paulo, Brazil; ^4^RNA Systems Biology Laboratory (RSBL), Departamento de Morfologia, Instituto de Ciências Biológicas, Universidade Federal de Minas Gerais (UFMG), Belo Horizonte, Brazil

**Keywords:** microRNA, autoantibodies, qRT-PCR, type 1 diabetes, miR-101-3p, miR-204-5p, autoimmunity

## Abstract

MicroRNAs (miRs) are master regulators of post-transcriptional gene expression, and they are often dysregulated in individuals suffering from diabetes. We investigated the roles of miR-101-3p and miR-204-5p, both of which negatively regulate insulin secretion and cell survival and are highly expressed in pancreatic β cells, in the context of type 1 diabetes (T1D) pathogenesis. Using quantitative real time PCR, we evaluated serum levels of miR-101-3p and miR-204-5p in four groups, including recent-onset T1D patients (T1D group; *n* = 50), individuals with normal glucose levels expressing one islet autoantibody (Ab) (single Ab group; *n* = 26) or multiple autoantibodies (multiple Ab group; *n* = 12), and healthy controls (control group; *n* = 43). An *in silico* analysis was performed to identify potential target genes of these miRNAs and to delineate enriched pathways. The relative expression of serum miR-101-3p was approximately three times higher in the multiple Ab and T1D groups than that in the single Ab and control groups (*p* < 0.0001). When considering all groups together, miR-101-3p expression was positively correlated with the level of islet autoantibodies GADA (*r* = 0.267; *p* = 0.0027) and IA-2A (*r* = 0.291; *p* = 0.001), and the expression of the miRNA was not correlated with levels of ZnT8A (*r* = 0.125; *p* = 0.183). miR-101-3p expression did not correlate with HbA1c (*r* = 0.178; *p* = 0.052) or glucose levels (*r* = 0.177; *p* = 0.051). No significant differences were observed in miR-204-5p expression among the analyzed groups. Computational analysis of the miR-101-3p target gene pathways indicated a potential activation of the HGF/c-Met, Ephrin receptor, and STAT3 signaling pathways. Our study demonstrated that the circulating levels of miR-101-3p are higher in T1D patients and in individuals with normal glucose levels, testing positive for multiple autoantibodies, indicating that miR-101-3p precedes loss of glucose homeostasis. The pathogenic role of miR-101-3p in T1D may involve multiple molecular pathways.

## Introduction

The loss of functional pancreatic β-cells is a key factor in the pathogenesis of both type 1 and type 2 diabetes (1, 2). Type 1 diabetes (T1D) is a heterogeneous autoimmune disease resulting from numerous mechanisms that lead to the destruction of insulin-producing pancreatic islet β cells and chronic hyperglycemia. Genetic factors, notably the immune-related HLA-DR3 and -DR4 alleles, and environmental factors, including chronic exposure to inflammatory cytokines, are involved in T1D pathophysiology (1, 2).

Islet autoantibodies are the most important humoral markers of immunological aggression against β-cells, and these markers are typically present in the preclinical phase of the disease and are important prognostic factors in the development of T1D. They include autoantibodies against insulin (IAA), glutamic acid decarboxylase 65 (GADA), tyrosine phosphatase (IA-2A), and zinc transporter 8 (ZnT8A). The presence of two or three autoantibodies has been demonstrated to confer a high risk for diabetes, estimated at ~85% over a 15-year period (2). It is difficult to define, however, exactly when and which individuals will progress to overt diabetes, thus justifying the search for other biomarkers to identify those who may benefit from preventive treatments.

The molecular and cellular mechanisms underlying progressive β cell destruction are not fully understood (1, 2). In the last 20 years, a new mechanism of post-transcriptional regulation of genes, performed by small RNAs of 21–25 non-single-stranded nucleotides called microRNAs (miRNAs or miRs), has been demonstrated to play a role in the “fine tuning” of gene expression in multiple biological processes and diseases (3)_._ miRs are involved in cell proliferation, differentiation, apoptosis, and metabolism (3), and their dysregulation is associated with several pathological conditions, including metabolic and autoimmune diseases such as T1D (3–5).

Roggli et al. (6) observed in NOD mice (an animal model of autoimmune diabetes) that the inflammatory cytokines IFN-γ, IL-1β, and TNFα increased the expression of miR-21, miR-34a, and miR-146a/b, all of which are related to β cell apoptosis, reduction of insulin secretion, and development of diabetes, highlighting their effect on the pathogenesis of diabetes.

Further, the progressive elevation of serum miR-375 (an abundant microRNA from islet cells) prior to the establishment of hyperglycemia in the NOD and C57BL/6 mice treated with streptozotocin (7) suggested its potential role as a circulating biomarker of β cell destruction.

The dysregulation of the miRNA profiles in T1D patients compared to those of healthy controls indicated the potential role of miRNAs as biomarkers of diabetes (8). Serum miRNA levels may mirror the destruction, dysfunction, or regeneration of β-cells and their immunological activity, likely through changes in the levels of miRNAs related to inflammation, β cell function and survival, and insulin secretion and sensitivity in the target cells (3). As serum miRNAs are stable and protected from degradation by RNase due to their inclusion in the microvesicles, exosomes, and apoptotic bodies, they may provide an appropriate tool to analyze pancreatic changes associated with T1D (9).

These data in the context of humans, however, are still controversial. Sebastiani et al. (10) demonstrated a positive correlation between miR-326 in peripheral blood lymphocytes and islet autoantibodies. Nielsen et al. (8) reported increased expression of several serum miRNAs related to apoptosis (miR-181a, miR-24, miR-210, miR-26a, miR-25) and β cell function (miR-148a, miR-24 miR-200a, miR-29a), while miR-25 was negatively correlated with residual function of β cells and positively correlated with glucose control. Liu et al. (11), who observed increased expression of other miRNAs (miR-1225-5p and miR-320) in recent –onset T1D patients, and Erener et al. (12), who reported five different miRNAs, were both unable to reproduce these data. Further, the altered expression of miRNAs in naïve CD4+T cell subsets derived from first-degree relatives of T1D patient presenting with two or more autoantibodies (13) was not confirmed in the serum samples of children at high risk for T1D development, a finding that was similar to that observed for healthy controls (14). It is also important to note that hyperglycemia acts as a complicating factor, as it is associated with the dysregulation of several miRNAs (15).

Pancreatic islets are rich in miR-101-3p (16). Zheng et al. (17) demonstrated the participation of miR-101a in IL-1β-mediated β cell dysfunction that resulted in decreased insulin content and increased β cell death. miR-101a was overexpressed in peripheral blood mononuclear cells (PBMC) obtained from patients suffering from long-term T1D (18). Increased serum levels of miR-101 positively correlated with HbA1c levels in patients diagnosed with type 2 diabetes (T2D) (19).

miR-204 is also highly expressed in human β cells (16), and its expression is high in murine models of T2D (20). Furthermore, miR-204 inhibits insulin transcription by targeting the transcription factor MAFA (v-maf avian musculoaponeurotic fibrosarcoma oncogene homolog A) (20). It also promotes β cell apoptosis induced by endoplasmic reticulum stress through regulation of the unfolded protein response-related protein kinase R-like ER kinase (PERK) (21). miR-204 levels increase with age and play a role in postnatal β cell maturation, and they also aid in the determination of the functional β cell mass in adult rats (22).

Given the possible relevance of miR-101-5p and miR-204-5p in reducing insulin secretion and β cell death, both of which are paramount events in early T1D pathogenesis, we analyzed their expression in serum samples from four clinical groups that included patients with recent-onset autoimmune diabetes, individuals without diabetes expressing single or multiple islet autoantibodies, and healthy controls. We intended to assess the expression of these markers in a phase that precedes the disease (islet antibody-positive subjects with preserved glucose metabolism) and during the early disease stage, a phase in which autoimmune β cell destruction process is still active. To provide new insights into the roles of the differentially expressed miRNAs in the pathogenesis of T1D, we performed pathway and functional enrichment analyses of putative target genes of the differentially expressed miRNAs.

## Materials and Methods

### Ethics and Consent

The study was approved by the Ethical Committee of Hospital das Clinicas HCFMUSP, Faculdade de Medicina, Universidade de São Paulo (Cappesq 11601), and we followed the guidelines of the Declaration of Helsinki. Informed consent was obtained from all patients, and serum samples were collected and stored at −80°C until use.

### Study Design and Patients

The study consisted of four clinical groups, including patients with recent-onset T1D ( ≤ 6 months from diagnosis—T1D group; *n* = 50) according to ADA criteria (23) with a mean age at diagnosis of 12.8 ± 6.5 years, individuals with normal blood glucose who tested positive for one islet autoantibody (single Ab group; *n* = 26) or multiple autoantibodies (multiple Ab group; *n* = 12), and healthy controls without a family history of inflammatory or autoimmune diseases (control group; *n* = 43). The demographic data and clinical characteristics of the selected individuals are described in Table 1.

**Table 1:** Demographic and clinical features of type 1 diabetes, islet autoantibody-positive and healthy control groups.

**Variable**	**Control group *n* = 43**	**Single Ab group *n* = 26**	**Multiple Ab group *n* = 12**	**T1D group *n* = 50**	***P-*value**
Glucose (mg/dL)	77 (74–83)	85 (78–88.5)	87 (80.75–94)	105 (77.5–201.5)^§^	0.0001
HbA1c (%)	5.5 (5.15–5.87)	5,25 (5.01–5.47)	5.2 (4.74–5.5)	7.9(6.45–9.12)^§^^,^ ^#^^,^ ^*^	<0.0001
IAA (nU/mL)	0 (0–20)	39.5 (0–126.30)*^£^*	101.5 (51.75–167.3)^β^, *^a^*		<0.0001
GAD65A (IU/mL)	0 (0–1.5)	3.75 (0–62.88)	228.8 (35.63–729.4)^β^, *^a^*	154(58.3–435.0)^§^^,^ ^#^	<0.0001
ZnT8Ab (IU/mL)	2.6 (0–6)	3.4 (1–17.6)	528.5 (7.82–888)^β^, *^a^*	498 (37–856)^§^^,^ ^#^	<0.0001
IA−2A (IU/mL)	1.8 (0–12.5)	0 (0–25)	168 (9–306.3)	567.5 (192.8–1936)^§^^,^ ^#^	<0.0001
C–peptide (ng/dL)	2.3 (1.7–3.0)	2 (1.1–2.8)	1.7 (1.1–3.2)	0.65 (0.48–1.20)^§^^,^ ^#^	<0.0001
Age (years)	15.82 ± 5.98	15.09 ± 8.00	15.96 ± 8.11	13.0 ± 6.52	0.115
Male/female	17/26	15/11	9/3	24/26	0.134
HLA–DR3 or DR4 alleles	29.03%	63.2%*^£^*	90.0%^β^, *^a^*	88.9%^§^	<0.0001

*Data are presented as median and inter-quartile range or mean ± standard deviation. Single Ab, individuals presenting one islet autoantibody; Multiple Ab, individuals presenting two or more islet autoantibodies; T1D, type 1 diabetes; HbA1c, glycated hemoglobin; IAA, insulin autoantibody (Normal value = ≤ 100 U/mL); GAD65A, glutamic acid decarboxylase autoantibody (NV ≤ 25 IU/mL); ZnT8A, zinc transporter-8 autoantibody (NV ≤ 16 IU/mL); IA-2A, tyrosine phosphatase autoantibody (NV ≤ 125 IU/mL)*.

§ T1D vs. Control p < 0.05;

# T1D vs. Single Ab p < 0.05;

* T1D × Multiple Ab p < 0.05;

β Multiple Ab × Single Ab p < 0.05;

a Multiple Ab × Control p < 0.05;

£ *Single Ab × Control p < 0.05*.

The four groups were matched by age (*p* = 0.12) and sex (*p* = 0.13). As expected, T1D patients exhibited higher levels of HbA1c and islet autoantibodies and lower levels of C-peptide (*p* < 0.0001) compared to those of the individuals in the control and single Ab groups. Glucose levels in TID patients were higher than those in controls (*p* = 0.0001). The multiple Ab group exhibited similar values to those of the T1D group for all variables with the exception of HbA1c levels, which were in the normal range. GADA and ZnT8A titers in the multiple Ab group were higher than those in the control and single Ab groups. IAA titer was higher in the single and multiple Ab groups than those in the control group.

The frequency of HLA-DR3 or -DR4 alleles was similar in the T1D and single and multiple Ab groups, and these were all higher than those in the control (*p* < 0.0001). The exclusion criteria included other types of diabetes (T2D, maturity onset diabetes of the young [MODY], and secondary diabetes), use of medications except insulin, or a febrile state within 10 days prior to blood collection.

### Assessment of Hemolysis

Serum samples hemolyzed during sample preparation can be contaminated by erythrocyte miRNAs. The degree of hemolysis in serum samples was assessed by spectrophotometry (Nanodrop 2000 spectrophotometer, Thermo Scientific, Waltham, Massachusetts, USA) measuring the absorbance at 350–650 nm. The degree of hemolysis was determined based on the optical density at 414 nm (absorbance peak of free hemoglobin), with additional peaks occurring at 541 and 576 nm that are indicative of a high degree of hemolysis. Samples were classified as “hemolyzed” if the OD_414_ exceeded a value of 0.2 (24). Previously hemolyzed samples were not used in the study.

### Extraction of Total Serum RNA Enriched in miRNAs

Total RNA enriched in miRNAs was isolated from serum samples (200 μL) using the miRNeasy Serum/Plasma kit (Qiagen, Hilden, Germany) according to the manufacturer's instructions. A synthetic miR, *Caenorhabditis elegans* (cel-miR-39) at 1.6 × 10^8^ copies/μL, was added as a spike-in control during the isolation to allow for technical normalization. The synthetic miRNA (10 pmol) was eluted with 300 μL of RNase-free water to form a stock solution of 2 × 10^10^ copies/μL. The stock solution was serially diluted with RNAse-free water to form the working solution of 1.6 × 10^8^ copies/μL that was added (3.5 μL) to each sample. Total RNA was eluted with 14 μL of RNase-free water. The concentration of the final material was determined by measuring the A_260_/A_280_ ratio using a NanoDrop ND-2000 apparatus (Thermo Scientific, Waltham, Massachusetts, USA). Samples of total RNA, including microRNAs, were stored at −80°C.

### Reverse Transcription and Quantitative Real-Time PCR

All samples were reverse transcribed and pre-amplified following the manufacturer's instructions. Briefly, a mixture of 10 μL of each of the TaqMan® MicroRNA Assays 5X primers (Thermo Scientific, USA) and hsa-miR-101-3p, hsa -miR-204-5p, and cel-miR-39 was used for the reverse transcription reaction to obtain the complementary DNA (cDNA). The reverse transcription reaction was performed in 0.2 mL tubes under cycling conditions that included 30 min at 16°C; 30 min at 42°C, and 5 min at 85°C in a Veriti® Thermal Cycler (Thermo Scientific, USA). Pre-amplification was performed after reverse transcription using 2.5 μL of the transcript product and 22.5 μL of the pre-amplification mix with cycling conditions that included 10 min at 95°C, 2 min at 55°C, and 2 min at 72°C, 12 cycles of 15 s at 95°C and 4 min at 60°C, and 10 min at 99.9°C. The quantitative real-time PCR (qRT-PCR) reaction mix for miRNAs contained hsa-miR-101-3p (assay ID 002253), hsa-miR-204-5p (assay ID 000058), Cel-miR-39 (assay ID 000200), 1.0 μL TaqMan® microRNA assay (20×), 1.0 μL pre-amplified RT product (cDNA), 5.0 μL TaqMan® Universal Master Mix II, No AmpErase® UNG (2×), and 3.0 μL nuclease-free water, with a final volume of 10 μL. qRT-PCR was performed in a 384-well plate, in duplicate, using the QuantStudio™ 12K Flex-(Thermo Scientific, Waltham, Massachusetts, USA). Cycling conditions included 10 min at 95°C, followed by 40 cycles of 15 s at 95°C and 60 s at 60°C, with the reaction ending at 4°C. The qRT-PCR data were presented as relative quantification using the comparative method as 2^−ΔΔ*Ct*^ for each miRNA (relative expression-fold change) as described (25). The control group was used as a calibrator in the 2^−ΔΔ*Ct*^ method for comparison between the T1D and Ab positive groups, and given this, the relative expression in each sample was calculated with respect to the mean expression value for healthy controls (adjusted value 1) and was presented as fold change. Fold change calculation for individual samples of the control group was calculated by obtaining the ΔΔCt value as performed for T1D samples and described in http://www.barbaradaviscenter.org.

Samples that did not amplify properly were excluded from the analyses. For miR-101-3p, there were one, four, and two samples in the T1D, single Ab, and control groups, respectively. For miR-204-5p, there were three, two, one, and three samples in the T1D, single Ab, control, and multiple Ab groups, respectively. Certain results were excluded because the duplicates were not adequate. This is a retrospective study that used a limited amount of serum/DNA for testing. For this reason, it was impossible to repeat the microRNA amplifications in a small number of samples. The same limitation was present for determinations of autoantibodies, glycemia, HbA1c, and HLA DR/DQ alleles. All data in this study are presented in Supplement 1. The total number of determinations for each variable in each group is presented in Supplement 2.

### Quantification of Glucose, Glycated Hemoglobin, and C-Peptide and Autoantibody Levels

Serum glucose levels were determined using an enzymatic colorimetric assay (LABTEST GOD-ANA, SP, Brazil), and glycated hemoglobin (HbA1c) levels were assessed by high performance liquid chromatography (HPLC). Fasting serum C-peptide levels were determined by radioimmunoassay (HCP-20K, Millipore Corporation, Billerica, MA, USA; normal values >0.5 ng/mL; intra- and inter-assay coefficients of variation (CV) were 4.5 and 9.3%, respectively). Serum levels of the autoantibodies IAA, GADA, and IA-2A were determined by radioimmunoassay (RSR limited, High Bentham, Lancaster, UK; CV <7%). The normal values for 700 healthy controls (3 standard deviations, SD) were <100, <25.0, and <125 IU/mL for IAA, GADA, and IA-2A, respectively. Serum levels of ZnT8A were measured by ELISA (KR770-96; Kronus, Boise, Idaho, USA; CV <7%). This assay detects and quantifies autoantibodies specific to residues R325 and W325 or to non-specific variants of residue 325. The normal value of ZnT8A in 321 healthy controls was defined as ≤ 16 IU/mL (3 SD).

### Pathway Analysis

Pathway analysis was performed using the Ingenuity Pathway Analysis (IPA) (Qiagen, Redwood City, California, USA) software which maintains a graphical database of networks of interacting genes including integrative information from literature, gene expression, and gene annotation. The lists of differentially expressed genes in patients with type 1 diabetes as compared to those antibody positive individuals and health controls were uploaded to IPA.

The IPA target filter tool, which relies on three algorithms (TargetScan, miRecords, and TarBase), was used to identify putative targets of miR-101-3p (based on the content of 2016-03 release), reviling several canonical pathways in diabetes. The list of experimentally validated targets of miR-101-3p was used to identify significantly (Fisher's exact test; *p* < 0.01) enriched canonical pathways within the miR-101-3p targets.

### Statistical Analysis

The variable distributions were verified by the Shapiro–Wilk normality test. Numerical variables with parametric and non-parametric distribution were analyzed by ANOVA and Kruskal–Wallis with Tukey's or Dunn's multiple comparison post-test, respectively, and they were adjusted for age, sex, and HbA1c levels. Correlations were performed using the Spearman correlation test. Qualitative variables were compared using the chi-square test or the Fisher's exact test using the statistical package GraphPad Prism (La Jolla, CA, USA) and JUMP 8 software (SAS, Cary, USA). Data were considered significant at *p* < 0.05.

## Results

**Figure 1:**
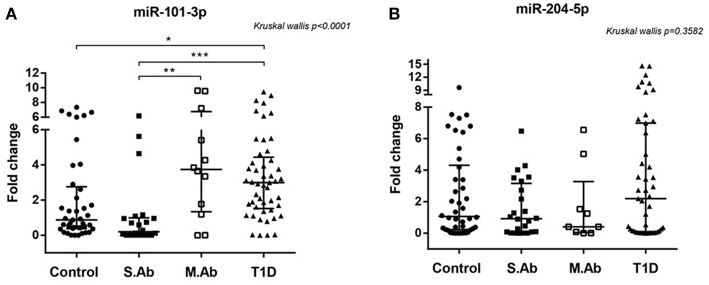
**(A)** Relative expression of serum miR-101-3p **(A)** in healthy controls (*n* = 41), individuals without diabetes expressing one single autoantibody (single Ab) (*n* = 22) or multiple autoantibodies (multiple Ab) (*n* = 12) and patients with recent type 1 diabetes (T1D) (*n* = 48). **(B)** Relative expression of serum miR-204-5p in healthy controls (*n* = 42), individuals without diabetes expressing a single autoantibody (single Ab) (*n* = 24) or multiple autoantibodies (multiple Ab) (*n* = 9), and patients with recent type 1 diabetes (T1D) (*n* = 46) ^*^p < 0.05, ^**^, ^***^p < 0.0001. Fold change: the qRT-PCR data were presented as relative quantification using the comparative method as 2^−ΔΔ*Ct*^ for each miRNA (relative expression-fold change) as described (25). The control group was used as a calibrator in the 2^−ΔΔ*Ct*^ method for comparison between the T1D and Ab positive groups, and therefore, the relative expression in each sample was calculated with respect to the mean expression value for healthy controls (adjusted value 1) and presented as fold change. Fold change calculation for individual samples of the control group was calculated by obtaining the ΔΔCt value as performed for T1D samples and described in http://www.barbaradaviscenter.org.

Circulating hsa-miR-101-3p was upregulated in T1D patients and non-diabetic individuals positive for two or more islet autoantibodies (multiple Ab group). Median serum levels of hsa-miR-101-3p were 3-fold or higher for both groups compared to levels in the control and single Ab groups (*p* < 0.0001; Figures 1A,B).

miR-101-3p levels in the patients of the multiple Ab group did not differ from those of the T1D group, and miR-101-3p levels in the patients of the single Ab group did not differ from those in controls (*p* > 0.05).

**Figure 2:**
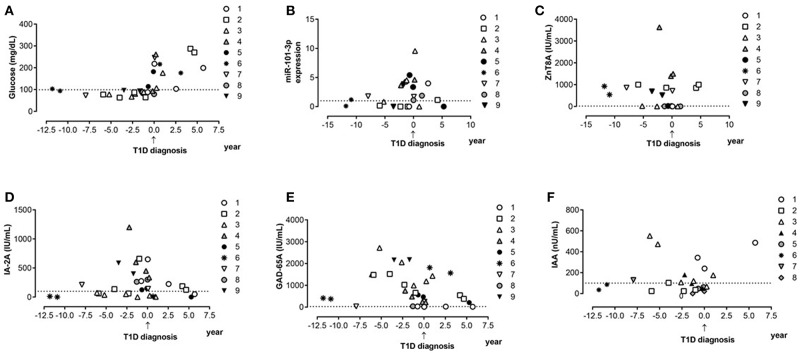
Progression to type 1 diabetes: miR-101-3p, islet autoantibodies, and glucose serum profile. Serum levels of glucose **(A)**, relative expression of miR-10-3p **(B)**, levels of autoantibodies against zinc transporter 8 (ZnT8A—Normal values-NV ≤ 16 IU/mL) **(C)**, tyrosine phosphatase (IA-2A—NV ≤ 125 IU/mL) **(D)**, glutamic acid 65 decarboxylase (GADA—NV ≤ 25 IU/mL) **(E)**, and insulin (IAA—NV ≤ 100 IU/mL) **(F)** before and after type 1 diabetes diagnosis. Each symbol type represents one patient over time (patient one to nine). Each individual was represented with the same symbol in the various graphs in this figure.

Using 1.0-fold change as the cutoff, we found that the number of subjects displaying upregulated serum miR-101-3p was similar between the control (43.9%) and single Ab (22.7%) groups, but lower than those of the multiple Ab (83.3%) and T1D (85.7%) groups (*p* < 0.0001), conferring odds ratios of 6.39 [confidence interval (CI): 1.24–32.91; *p* = 0.02] and 7.67 (CI: 2.79–21.06; *p* < 0.001) for multiple Ab and T1D groups, respectively, relative to control group, and odds ratios of 17 (2.76–104.6) and 20.4 (5.68–73.28), respectively, relative to single Ab group. The presence of up-regulated serum miR-101−3p or multiple autoantibodies conferred similar risk for diabetes (70 × 75%; OR = 0.78, CI = 0.12–5.1; *p* = 1.0). The data of nine multiple Ab individuals (from total of 12) who evolved to T1D are presented in Figures 2A–F. The levels of miR-101-3p and the autoantibodies significantly increased prior to the time of diagnosis.

The frequency of HLA-DR3 and -DR4 alleles was lower in the control group than in the other three groups. It was not possible to establish the role of these alleles in the expression of miR-101-3p and miR-204-5p.

In contrast, miR-204-5p was not differentially expressed among any of the tested groups. Despite the lack of significant differences, the relative expression of miR-101-3p positively correlated with that of miR-204-5p in the control (*r* = 0.374; *p* = 0.018) and single Ab (*r* = 0.502; *p* = 0.024) groups, but not in the multiple Ab (*r* = −0.07; *p* = 0.880) and T1D (*r* = 0.141; *p* = 0.350) groups.

### Correlation of hsa*-*miR-101-3p Expression With T1D Pathophysiological Parameters

**Figure 3:**
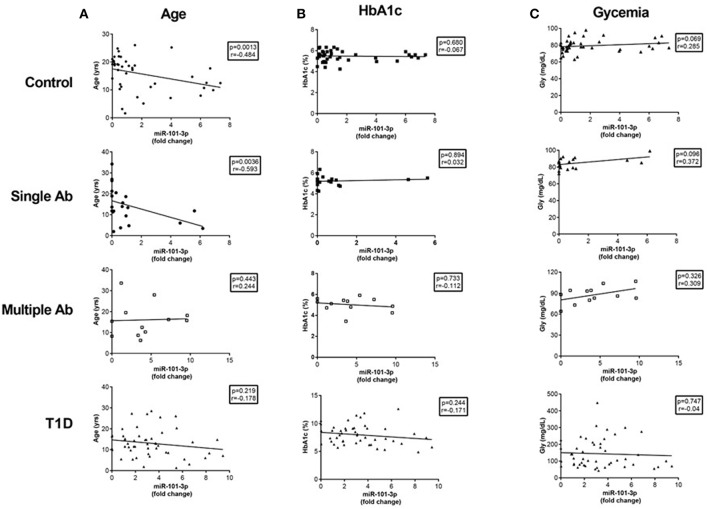
Correlations of serum miR-101-3p relative expression with age **(A)** and levels of glycated hemoglobin (HbA1c) **(B)** and glucose **(C)** levels in healthy controls, individuals without diabetes expressing one single autoantibody (single Ab) or multiple autoantibodies (multiple Ab), and patients with recent type 1 diabetes (T1D). The number of determinations related to age, HbA1c, and glucose levels are presented in parenthesis for healthy controls (*n* = 41, 39, 41), single Ab (*n* = 22, 20, 21), multiple Ab (*n* = 12, 12, 12), and T1D groups (*n* = 48, 47, 47). Fold change: the qRT-PCR data were presented as relative quantification using the comparative method as 2^−ΔΔ*Ct*^ for each miRNA (relative expression-fold change) as described (25). The control group was used as a calibrator in the 2^−ΔΔ*Ct*^ method for comparison between the T1D and Ab positive groups, and therefore, the relative expression in each sample was calculated with respect to the mean expression value for healthy controls (adjusted value 1) and was presented as fold change. Fold change calculation for individual samples of the control group was determined by obtaining the ΔΔCt value as performed for T1D samples and described in http://www.barbaradaviscenter.org.

No correlation in the expression of miR-101-3p and miR-204-5p was found in regard to HbA1c and glucose levels for any group (Figures 3A–C). A negative correlation between hsa-miR-101-3p and age was observed only in the control (*r* = −0,484; *p* = 0.001) and single Ab (*r* = −0.593; *p* = 0.004.) groups, and not in the multiple Ab and T1D groups.

No correlation existed between miR-101-3p and age at diagnosis (*r* = −0.163; *p* = 0.261) or between miR-101-3p and the duration of diabetes (*r* = 0.086; *p* = 0.559).

**Figure 4:**
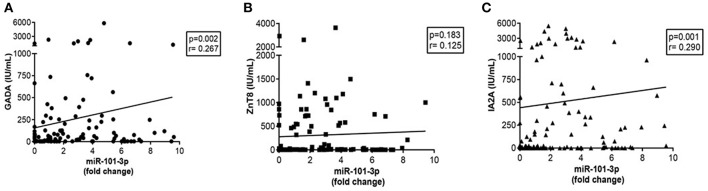
Correlations of serum miR-101-3p relative expression with autoantibodies: anti-glutamic acid decarboxylase GADA (NV ≤ 25 IU/mL) (*n* = 123) **(A)**, anti-zinc transporter 8- ZnT8A (Normal values-NV ≤ 16 IU/mL) (*n* = 115) **(B)**, and anti-tyrosine phosphatase IA-2A (NV ≤ 125 IU/mL) (*n* = 121) **(C)** in the entire cohort. Fold change: the qRT-PCR data were presented as relative quantification using the comparative method as 2^−ΔΔ*Ct*^ for each miRNA (relative expression-fold change) as described (25). The control group was used as a calibrator in the 2^−ΔΔ*Ct*^ method for comparison between the T1D and Ab positive groups, and therefore, the relative expression in each sample was calculated with respect to the mean expression value for healthy controls (adjusted value 1) and was presented as fold change. Fold change calculation for individual samples of the control group was determined by obtaining the ΔΔCt value as performed for T1D samples and described in http://www.barbaradaviscenter.org.

For all groups, miR-101-3p expression was positively correlated with the levels of GADA (*r* = 0.267; *p* = 0.0027) and IA-2A (*r* = 0.291; *p* = 0.001), but not with the levels of ZnT8A (*r* = 0.125; *p* = 0.183; Figures 4A–C). There was no correlation of miR-101-3p with HbA1c (*r* = 0.178; *p* = 0.052 or glucose levels (*r* = 0.177; *p* = 0.051), and miR-204-5p levels correlated with miR-101-3p (*r* = 0.271; *p* = 0.003) in the entire cohort.

### Potential Targets and Pathways Regulated by miR-101-3p in T1D

**Figure 5:**
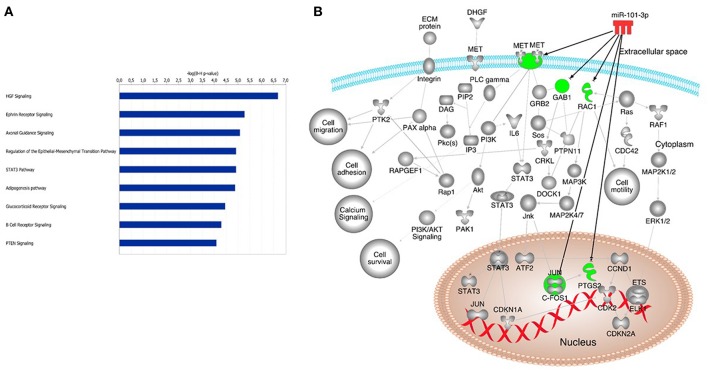
Potential canonical pathways enriched in autoimmune type 1 diabetes. Canonical pathways of putative miR-101-3p targets **(A)**. Additional pathways that may be associated with type 1 diabetes pathogenesis; **(B)** Hepatocyte growth factor (HGF); MET proto-oncogene (HGF receptor); Growth factor receptor bound protein 2 associated binding protein 1 (GAB1); Ras-related C3 botulinum toxin substrate 1 (RAC1); prostaglandin-endoperoxide synthase 2 (COX2); Fos proto-oncogene (c-FOS); Ephrin receptor signaling (Eph), STAT3 (Signal transducer and activator of transcription pathway).

Using the IPA software, we identified a total of 726 putative miR-101-3p targets based on miRNA-target relationships that have been experimentally validated. Canonical pathway analysis of the list of 726 putative hsa-miR-101-3p targets was performed. The most significantly enriched canonical pathways are listed in Figures 5A,B. We observed hepatocyte growth factor (HGF) signaling as the most enriched canonical pathway. The hsa-miR-101-3p targets within the HGF signaling pathway are HGF, MET proto-oncogene (HGF receptor), growth factor receptor-bound protein 2-associated binding protein 1 (GAB1), Ras-related C3 botulinum toxin substrate 1 (RAC1), prostaglandin-endoperoxide synthase 2 (COX2), and Fos proto-oncogene (c-FOS) (Figures 5A,B). Additional enriched pathways that may be pathogenetically relevant in T1D include the ephrin receptor signaling pathway, involved in glucose sensing and insulin production by pancreatic β cells, and the STAT3 (Signal Transducer and Activator of Transcription) pathway, involved in β cell survival. The experimentally validated molecules in these signaling pathways (*p* < 0.01) are presented in Supplement 3.

## Discussion

In this study, we found that serum levels of miR-101-3p were higher (3-fold or more) in the recent-onset patients with T1D than in the control and single Ab groups. Similarly, patients who tested positive for two or more autoantibodies exhibited miR-101-3p levels similar to T1D patients, and these were significantly higher than those in the patients in the control and single Ab groups. No significant difference was observed between the single Ab and control groups. Further, the frequency of subjects displaying upregulated levels of serum miR-101-3p increased from healthy controls and single Ab individuals to individuals with Multiple Ab and overt T1D, and these findings were in agreement with the degree of β cell aggression. The positive correlation of miR-101-3p with GADA and IA-2A levels reinforces our data.

The glucose, HbA1c, and C-peptide levels were comparable between the multiple Ab and control groups, indicating preserved β cell function. Among multiple Ab individuals, miR-101-3p levels increased prior to and at the time of T1D diagnosis. No significant differences were found in regard to miR-204-5p levels among the analyzed groups, and there was no correlation of miR-204-5p levels with metabolic parameters.

As expected, those groups presenting single or multiple Ab and individuals suffering from T1D exhibited a greater frequency of the HLA alleles that are usually associated with autoimmunity (HLA-DR3 and -DR4) (2); however, it was not possible to establish its role in the expression of miR-101-3p and miR-204-5p.

*In silico* pathway analysis indicated that miR-101-3p targets the c-Met-HGF, ephrin receptor, and STAT3 pathways associated with insulin production, survival, and death of pancreatic β cells (26).

Our study analyzed four distinct groups of individuals, where three of these groups were at different stages of β cell aggression (recent-onset T1D patients and individuals without diabetes expressing one or multiple islet autoantibodies) and one consisted of healthy controls. Although there are previous studies regarding circulating microRNA profiling (8, 10–12, 15), our study was the first to find increased levels of miR-101-3p in the serum of T1D patients. This possible discrepancy with previous studies may have been due to several factors, including the number and age of patients with recent-onset T1D, the duration of diabetes, or different ethnicities among samples (Canada vs. Brazil). Increased miR-101 levels were found in PBMC (18) and naïve CD4+ T cells (13) obtained from late-stage adult T1D patients, indicating a potential effect on the pathophysiology of T1D in humans that is in accordance with the findings of a study performed in animals (17).

There was a negative correlation of miR-101-3p levels with age and a positive correlation with miR-204-5p levels among healthy controls and single Ab individuals. These correlations were not observed in the multiple Ab and T1D groups. The negative correlation with age suggests that miR-101-3p levels may be associated with a greater susceptibility of the pancreas to injury or to the greater turnover and remodeling of β cells of the young children (27). Changes in the level of specific miRNAs that occur during aging appear to affect the proliferative capacity of rat β cells, ultimately interfering with their ability to expand (28).

We found that miR-101-3p levels in patients of the multiple Ab group were similar to those of the T1D group and higher than those of the control and single Ab groups. This indicates that miR-101-3p levels were also increased in the group exhibiting a high risk of developing T1D (2). Indeed, nine of these patients developed T1D in a follow-up of 0.7–11 years. The possible role of miR-101-3p in the pathogenesis of T1D is also corroborated by the increase in the number of subjects with multiple Ab or T1D displaying upregulated serum miR-101-3p and the positive correlation of miR-101-3p with GADA and IA-2A levels. Therefore, miR-101-3p could be associated with disease activity/β cell damage in individuals exhibiting normal glucose levels.

The failure to observe a correlation between circulating miR-101-3p and HbA1c or glucose levels in T1D contradicts previous findings regarding type 2 diabetes (19) and with the observation that *in vitro* exposure to high glucose induces miR-101 expression in endothelial cells (29). Although the latter suggests that increased glucose may be one of the stimuli for miR-101 tissue expression, our results suggest that elevated serum miR-101-3p levels in T1D and multiple autoantibody-positive subjects were independent of metabolic control. Specifically, the finding of normal Hb1Ac and glucose levels in subjects expressing two or more autoantibodies without diabetes indicates that miR-101-3p increase precedes T1D progression.

The cellular origin of the increased levels of circulating miR-101-3p in T1D and autoantibody-positive patients is unclear. The direct upregulation of miR-101 by IL-1β in MIN6 cells involves the role of inflammatory cytokine signaling in increasing miR-101 levels (17). The finding of increased levels during the time of T1D development (Figure 2) suggests that miR-101 is released from injured and dying β cells, as it is known that the cytokine IL-1 β directly increases miR-101 expression in β cells (17). However, miR-101 is also expressed in immune cells (13, 18, 30), where it appears to favor inflammatory cytokine production (30–32) associated with reduced antigen-specific regulatory T cell function and loss of immune tolerance (33), ultimately promoting T1D-associated inflammation. Additional targets that potentially lead to reduced β cell mass and insulin secretion include Onecut2 transcription factor (17), Ephrins and Ephrin receptors (34), and the antiapoptotic HGF/c-Met and STAT3 signaling (35–42).

Additionally, miR-101 expression is also upregulated in other autoimmune diseases, such as lupus erythematosus (43), multiple sclerosis (44), transplant rejection (45), and inflammatory bowel disease (46, 47).

Our study does, however, include limitations. The sample sizes were relatively small, the study was single-centered, and some predicted gene targets have not yet been experimentally validated in pancreatic β cells. Quantification of miRNAs is hampered by low RNA yield in serum. Inherent difficulties of this new methodology, lack of standardized sample collection and processing procedures, and analytical approaches using reference gene or global normalization should be considered for future studies.

Our study demonstrating increased levels of miR-101-3p in early-onset diabetes and euglycemic autoantibody-positive patients is consistent with several literature reports on its deleterious effects on pancreatic β cell insulin secretion, glucose sensing, and increasing β cell susceptibility to inflammatory cytokine-induced apoptosis. Additionally, this miRNA has been shown to increase the inflammatory potential of immune cells. It can thus be hypothesized that systemic exposure to miR-101-3p, such as that observed in recent-onset T1D patients and multiple islet autoantibody-positive subjects without diabetes, may fuel disease progression at both the effector and target sides of pathogenesis. As a corollary, systemic miR-101-3p antagonism could reduce inflammation and increase β cell survival and function by acting at multiple sites, and thus may possess therapeutic potential for recent-onset T1D.

## Ethics Statement

The study was approved by the Ethical Committee of Hospital das Clinicas HCFMUSP, Faculdade de Medicina, Universidade de São Paulo (Cappesq 11601), and followed guidelines of the Declaration of Helsinki. Informed consent was obtained from the patients and serum samples were collected and stored at −80°C until use.

## Author Contributions

AS, EC, and MS designed the research and finalized the manuscript. AS and LF conducted the research. All authors contributed to the analysis of the data and reviewed the manuscript.

### Conflict of Interest Statement

The authors declare that the research was conducted in the absence of any commercial or financial relationships that could be construed as a potential conflict of interest.
