# Supplementary material for: Increased Expression of Circulating microRNA 101-3p in Type 1 Diabetes Patients: New Insights Into miRNA-Regulated Pathophysiological Pathways for Type 1 Diabetes
Source: Front Immunol. 2019 Jul 23;10:1637. doi: 10.3389/fimmu.2019.01637 (PMC6665278; doi:10.3389/fimmu.2019.01637)
Supplement: Supplementary file 2 [file Data_Sheet_2.docx]

**Supplement 2: Number of determinations of each variable in the four study groups**

| Variables |  |  | Groups |  |
| --- | --- | --- | --- | --- |
|  | Control | Single Antibody | Multiple Antibodies | Type 1 diabetes |
| miR-101-3p (relative expression ) | 41 | 22 | 12 | 48 |
| miR-204-5p (relative expression ) | 42 | 24 | 9 | 46 |
| glucose (mg/dL) | 43 | 25 | 12 | 48 |
| HbA1c (%) | 41 | 24 | 12 | 48 |
| IAA (IU/mL) | 31 | 24 | 12 |  |
| GADA (IU/mL) | 43 | 26 | 12 | 49 |
| ZnT8A (IU/mL) | 42 | 21 | 10 | 47 |
| IA2A (IU/mL) | 43 | 26 | 10 | 49 |
| Age (years) | 43 | 26 | 12 | 49 |
| age at diagnosis (Years) |  |  |  | 49 |
| duration of diabetes (Years) |  |  |  | 49 |
| C-Peptide (ng/mL) | 35 | 17 | 5 | 25 |
| HLA-DRB1 allele | 31 | 17 | 10 | 45 |
| HLA-DRB1 allele | 31 | 17 | 10 | 45 |
| HLA-DQB1 allele | 31 | 16 | 10 | 45 |
| HLA-DQB1 allele | 31 | 16 | 10 | 45 |
| HLA DR/DQ Haplotypes | 31 | 16 | 10 | 45 |
| HLA DR/DQ Haplotypes | 31 | 16 | 10 | 45 |

HbA1c= Glicated hemoglobina; IAA= insulin autoantibody; GAD= glutamic acid descarboxilase autoantibody; IA2A= tyrosine phosphatase autoantibody
