# Supplementary material for: Increased Expression of Circulating microRNA 101-3p in Type 1 Diabetes Patients: New Insights Into miRNA-Regulated Pathophysiological Pathways for Type 1 Diabetes
Source: Front Immunol. 2019 Jul 23;10:1637. doi: 10.3389/fimmu.2019.01637 (PMC6665278; doi:10.3389/fimmu.2019.01637)
Supplement: Supplementary file 3 [file Data_Sheet_3.pdf]

Supplement 3- The experimentally validated molecules in signaling pathways of miR-101-3p

1-Genes of Hepatocyte Receptor Signaling Pathway

Symbol	Location	Family	Gene ID for Human
AKT	Cytoplasm	group	
AP-1	Nucleus	complex	
ATF2	Nucleus	transcription regulator	1386
c-FOS	Nucleus	transcription regulator	2353
c-JUN	Nucleus	transcription regulator	3725
c-RAF	Cytoplasm	kinase	5894
C3G	Cytoplasm	other	2889
CDC42	Cytoplasm	enzyme	998
CDK2	Nucleus	kinase	1017
COX2	Cytoplasm	enzyme	5743
CRKL	Cytoplasm	kinase	1399
CYCLIN D1	Nucleus	transcription regulator	595
DOCK1	Cytoplasm	other	1793
ELK-1	Nucleus	transcription regulator	2002
ERK1/2	Cytoplasm	group	
ETS	Nucleus	group	
ETS-ELK1	Nucleus	complex	
FAK	Cytoplasm	kinase	5747
GAB1	Cytoplasm	kinase	2549
GRB2	Cytoplasm	kinase	2885
HGF	Extracellular Space	growth factor	3082
IL-6	Extracellular Space	cytokine	3569
Integrin	Plasma Membrane	complex	
JNK	Cytoplasm	group	
MEK1/2	Cytoplasm	group	
MEKK	Cytoplasm	group	
MET	Plasma Membrane	kinase	4233
Met dimer	Plasma Membrane	complex	
MKK4/7	Cytoplasm	group	
p16INK4a	Nucleus	transcription regulator	1029
p21CIP1	Nucleus	kinase	1026
PAK1	Cytoplasm	kinase	5058
PI3K	Cytoplasm	complex	
PKC	Cytoplasm	group	
PLCγ	Cytoplasm	group	
PXN	Cytoplasm	other	5829
RAC1	Plasma Membrane	enzyme	5879
RAP1	Cytoplasm	group	
RAS	Cytoplasm	group	
SHP2	Cytoplasm	phosphatase	5781
SOS	Cytoplasm	group	
STAT3	Nucleus	transcription regulator	6774

Stat3 dimer	Nucleus	complex

2- Genes of Ephrin Receptor Signaling Pathway

Symbol	Location	Family	Gene ID for Human
ABI-1	Cytoplasm	other	10006
ABL	Nucleus	kinase	25
ADAM10	Plasma Membrane	peptidase	102
AKT	Cytoplasm	group	
ANGPT1	Extracellular Space	growth factor	284
ARP2/3	Cytoplasm	complex	
AXIN	Cytoplasm	other	8312
c-RAF	Cytoplasm	kinase	5894
C3G	Cytoplasm	other	2889
CAP	Plasma Membrane	other	10580
CAS	Plasma Membrane	enzyme	9564
CDC42	Cytoplasm	enzyme	998
CFL	Cytoplasm	group	
CREB	Nucleus	group	
CRK	Cytoplasm	group	
CXCR4	Plasma Membrane	G-protein coupled receptor	7852
EFNA	Plasma Membrane	group	
Efna dimer	Plasma Membrane	complex	
EFNB	Plasma Membrane	group	
Efnb dimer	Plasma Membrane	complex	
EGF	Extracellular Space	growth factor	1950
EPHA	Plasma Membrane	group	
Epha dimer	Plasma Membrane	complex	
EPHB	Plasma Membrane	group	
Ephb dimer	Plasma Membrane	complex	
ERK1/2	Cytoplasm	group	
FAK	Cytoplasm	kinase	5747
FGF1	Extracellular Space	growth factor	2246
FYN	Plasma Membrane	kinase	2534
G alpha-G beta-GDP-G gamma	Cytoplasm	complex	
Gα	Cytoplasm	group	
Gβ	Cytoplasm	group	
Gγ	Cytoplasm	group	
GRB2	Cytoplasm	kinase	2885
GRB4	Cytoplasm	kinase	8440
GRIN	Plasma Membrane	complex	
Integrin	Plasma Membrane	complex	
ITSN1	Cytoplasm	other	6453
JAK2	Cytoplasm	kinase	3717
KALRN	Cytoplasm	kinase	8997
LIMK	Cytoplasm	group	
LMW-PTP	Cytoplasm	phosphatase	52

MEK1/2	Cytoplasm	group	
NCK	Cytoplasm	kinase	4690
Nck1-Nik	Cytoplasm	complex	
NGEF	Cytoplasm	other	25791
NIK	Cytoplasm	group	
p62DOK	Plasma Membrane	kinase	1796
PAK	Cytoplasm	group	
PDGF	Extracellular Space	complex	
PI3Kγ	Cytoplasm	group	
PTP-BL	Cytoplasm	phosphatase	5783
PXN	Cytoplasm	other	5829
RAC	Cytoplasm	group	
RAP1	Cytoplasm	group	
RAS	Cytoplasm	group	
RAS GAP	Cytoplasm	transporter	5921
RGS3	Nucleus	other	5998
RHOA	Cytoplasm	enzyme	387
ROCK	Cytoplasm	group	
SDC2	Plasma Membrane	other	6383
SDCBP	Plasma Membrane	enzyme	6386
SDF-1	Extracellular Space	cytokine	6387
SHC	Cytoplasm	other	6464
SHEP1	Cytoplasm	other	10044
SHP2	Cytoplasm	phosphatase	5781
SOS	Cytoplasm	group	
SRC	Cytoplasm	kinase	6714
STAT3	Nucleus	transcription regulator	6774
VEGF	Extracellular Space	group	
VSM-RHOGEF	Cytoplasm	other	22899
WASP	Cytoplasm	group	

3-Genes of Axonal Guidance Signaling Pathway

Symbol	Location	Family	Gene ID for Human
ABL	Nucleus	Kinase	25
ABLIM	Cytoplasm	Group	
ADAM10	Plasma Membrane	Peptidase	102
AKT	Cytoplasm	Group	
ARHGEF11	Cytoplasm	Other	9826
ARHGEF12	Cytoplasm	Other	23365
ARHGEF6/7	Cytoplasm	Group	
ARP2/3	Cytoplasm	Complex	
Axonbranching	Other	Function	
Axondetachment	Other	Function	
Axondynamics	Other	Function	
Axonrepulsion	Other	Function	
Axon attraction	Other	Function	
Axon guidance	Other	Function	
Axon migration	Other	Function	
Axon outgrowth	Other	Function	
Axon repulsion	Other	Function	
Axon turningresponse	Other	Function	
BAIAP2	Plasma Membrane	Kinase	10458
BDNF	Extracellular Space	growth factor	627
BMP	Extracellular Space	Group	
BMP7	Extracellular Space	growth factor	655
Bmp7-Gdf7	Extracellular Space	Complex	
c-Raf	Cytoplasm	Kinase	5894
Calcineurin	Cytoplasm	Complex	
CAS	Plasma Membrane	Enzyme	9564
CDC42	Cytoplasm	Enzyme	998
CDK5	Nucleus	Kinase	1020
CFL	Cytoplasm	Group	
COS2	Extracellular Space	Other	374654
CRAM	Cytoplasm	Enzyme	56896
CRK	Cytoplasm	Group	
CRMP2	Cytoplasm	Enzyme	1808
CRMP2-KLC1-Tubulin	Cytoplasm	Complex	
CXCR4	Plasma Membrane	G-protein coupled receptor	7852
DCC	Plasma Membrane	transmembrane receptor	1630
Dcc dimer	Plasma Membrane	Complex	
Dcc-Unc5	Plasma Membrane	Complex	
DOCK1	Cytoplasm	Other	1793
Dock1-Pak	Cytoplasm	Complex	
EFNA	Plasma Membrane	Group	

Efna dimer	Plasma Membrane	Complex	
EFNB	Plasma Membrane	Group	
Efnb dimer	Plasma Membrane	Complex	
EGF	Extracellular Space	growth factor	1950
eIF4E	Cytoplasm	translation regulator	1977
EPHA/B	Plasma Membrane	Group	
EPHA	Plasma Membrane	Group	
Epha dimer	Plasma Membrane	Complex	
EphA/B dimer	Plasma Membrane	Complex	
EPHB	Plasma Membrane	Group	
Ephb dimer	Plasma Membrane	Complex	
ERBB2	Plasma Membrane	Kinase	2064
ERK1/2	Cytoplasm	Group	
FAK	Cytoplasm	Kinase	5747
FARP2	Cytoplasm	Other	9855
FES	Cytoplasm	Kinase	2242
FU	Cytoplasm	Kinase	27148
FYN	Plasma Membrane	Kinase	2534
FZD	Plasma Membrane	Group	
G alpha-G beta-GDP-G gamma	Cytoplasm	Complex	
Gα	Cytoplasm	Group	
Gαi	Cytoplasm	Group	
Gβ	Cytoplasm	Group	
Gγ	Cytoplasm	Group	
GDF7	Extracellular Space	growth factor	151449
GDP	Other	chemical-endogenous mammalian	
GIT1	Nucleus	Kinase	28964
GLI	Cytoplasm	Group	
Gli-Kif7-Stk36-Sufu	Cytoplasm	Complex	
GRB2	Cytoplasm	Kinase	2885
GRB4	Cytoplasm	Kinase	8440
GSK3β	Nucleus	Kinase	2932
GTP	Other	chemical-endogenous mammalian	
HHIP	Plasma Membrane	Other	64399
IGF-1	Extracellular Space	growth factor	3479
Integrin	Plasma Membrane	Complex	
Intersectin	Cytoplasm	Other	6453
IP3	Other	chemical-endogenous mammalian	
ITGB1	Plasma Membrane	transmembrane receptor	3688
KALRN	Cytoplasm	Kinase	8997
KLC1	Cytoplasm	Other	3831
L1CAM	Plasma Membrane	Other	3897
LIMK	Cytoplasm	Group	
LINGO1	Plasma Membrane	Other	84894
MAG	Plasma Membrane	Other	4099
MEK1/2	Cytoplasm	Group	

MET	Plasma Membrane	Kinase	4233
Metalloproteinase	Extracellular Space	Group	
MICAL	Cytoplasm	Enzyme	64780
MLC	Cytoplasm	Group	
MNK1	Cytoplasm	Kinase	8569
NCK1	Cytoplasm	Kinase	4690
Netrin	Extracellular Space	Group	
Netrin G	Extracellular Space	Other	22854
NFAT	Cytoplasm	Complex	
NGEF	Cytoplasm	Other	25791
NGF	Extracellular Space	growth factor	4803
NGFR	Plasma Membrane	transmembrane receptor	4804
NGL1	Plasma Membrane	Other	57689
NGR	Plasma Membrane	transmembrane receptor	65078
NOGO	Cytoplasm	Other	57142
NRP	Plasma Membrane	Group	
Nrp-PlexinA	Plasma Membrane	Complex	
NT3/4	Extracellular Space	Group	
Ntrk dimer	Plasma Membrane	Complex	
PAK	Cytoplasm	Group	
PDGF	Extracellular Space	Complex	
PFN	Cytoplasm	Group	
PI3K	Cytoplasm	Complex	
PI3K p110	Cytoplasm	Group	
PI3K p85	Cytoplasm	Group	
PKA	Cytoplasm	Complex	
PKC	Cytoplasm	Group	
PLC	Cytoplasm	Group	
Plexin A	Plasma Membrane	Group	
Plexin B3	Plasma Membrane	transmembrane receptor	5365
Plexin C	Plasma Membrane	transmembrane receptor	10154
PLXNB1	Plasma Membrane	Group	
PLXND1	Plasma Membrane	transmembrane receptor	23129
PLXND1	Plasma Membrane	Group	
PXN	Cytoplasm	Other	5829
R-Ras	Cytoplasm	Enzyme	6237
RAC	Cytoplasm	group	
RAP1	Cytoplasm	group	
RAPL	Plasma Membrane	other	83593
Ras	Cytoplasm	group	
RASGAP	Cytoplasm	transporter	5921
RGS3	Nucleus	other	5998
RHOA	Cytoplasm	enzyme	387
RHOD	Cytoplasm	enzyme	29984
RND1	Cytoplasm	enzyme	27289
ROBO 1/2	Plasma Membrane	group	

ROBO 3	Plasma Membrane	transmembrane receptor	64221
ROCK	Cytoplasm	group	
SDC2	Plasma Membrane	other	6383
SDCBP	Plasma Membrane	enzyme	6386
SDF-1	Extracellular Space	cytokine	6387
SEMA3	Extracellular Space	group	
SEMA4	Plasma Membrane	group	
SEMA5A	Plasma Membrane	transmembrane receptor	9037
SEMA6	Plasma Membrane	group	
SEMA7A	Plasma Membrane	transmembrane receptor	8482
Shank	Plasma Membrane	other	22941
SHC	Cytoplasm	other	6464
SHEP1	Cytoplasm	enzyme	8924
Shh	Extracellular Space	peptidase	6469
SHP2	Cytoplasm	phosphatase	5781
SLIT	Extracellular Space	group	
SMO	Plasma Membrane	G-protein coupled receptor	6608
SOS	Cytoplasm	group	
srGAP	Cytoplasm	group	
SUFU	Nucleus	transcription regulator	51684
TRK	Plasma Membrane	group	
Tubulin	Cytoplasm	complex	
UNC5	Plasma Membrane	group	
VASP	Plasma Membrane	other	7408
VEGF	Extracellular Space	group	
VSM-RHOGEF	Cytoplasm	other	22899
WASP	Cytoplasm	group	
Wnt	Extracellular Space	group	
Wnt5a	Extracellular Space	cytokine	7474

4- Genes of Regulation of the Epithelial Mesenchymal Transition Pathway

Symbol	Location	Family	Gene ID for Human
β-Catenin	Nucleus	transcription regulator	1499
β-catenin-APC-AXIN-GSK3β	Other	complex	
β-catenin-BCL9-PYGO-TCF/LEF	Other	complex	
γ-Secretase	Plasma Membrane	complex	
AKT	Cytoplasm	group	
APC	Nucleus	enzyme	324
AXIN	Cytoplasm	other	8312
BCL9	Nucleus	other	607
c-Met	Plasma Membrane	kinase	4233
Claudin-3	Plasma Membrane	transmembrane receptor	1365
CSL	Nucleus	transcription regulator	3516
CSL-HIF1A-MAML1-NICD	Other	complex	
DVL	Cytoplasm	group	
E-Cadherin	Plasma Membrane	other	999
E2A	Nucleus	transcription regulator	6929
EGF	Extracellular Space	growth factor	1950
EGFR	Plasma Membrane	kinase	1956
EGR1	Nucleus	transcription regulator	1958
ERK1/2	Cytoplasm	group	
ESRP	Nucleus	other	80004
ETS1	Nucleus	transcription regulator	2113
FGF	Extracellular Space	group	
FGF dimer	Other	complex	
FGF18/20	Other	group	
FGFR	Plasma Membrane	group	
FOXC2	Nucleus	transcription regulator	2303
Frizzled	Plasma Membrane	group	
FRS2	Plasma Membrane	kinase	10818
FRS2-GRB2-SHP2	Other	complex	
GAB1	Cytoplasm	kinase	2549
GRB2	Cytoplasm	kinase	2885
GSC	Nucleus	transcription regulator	145258
GSK3β	Nucleus	kinase	2932
HGF	Extracellular Space	growth factor	3082
HIF1A	Nucleus	transcription regulator	3091
HMGA2	Nucleus	enzyme	8091
Hypoxia	Other	disease	
ID2	Nucleus	transcription regulator	3398
JAGGED-1/2	Other	group	
JAK	Cytoplasm	group	
LOX	Extracellular Space	enzyme	4015
MAML1	Nucleus	transcription regulator	9794

MEK	Cytoplasm	group	
miR-155	Cytoplasm	microRNA	406947
miR-192	Cytoplasm	microRNA	406997|406967
miR-200b	Cytoplasm	microRNA	406984|406933|406983|554210|406985
miR-34a	Cytoplasm	microRNA	407041|407040|407042
MMP-2	Extracellular Space	peptidase	4313
MMP-9	Extracellular Space	peptidase	4318
N-Cadherin	Other	group	
NF-κB	Nucleus	complex	
NOTCH	Plasma Membrane	transcription regulator	4851
NOTCH	Plasma Membrane	group	
PAR6	Other	group	
PDGF-D	Extracellular Space	growth factor	80310
PDGF-D dimer	Extracellular Space	complex	
PDGF-Rβ	Plasma Membrane	kinase	5159
PDGF-Rβ dimer	Other	complex	
PI3K	Cytoplasm	complex	
PYGO	Other	group	
Raf	Cytoplasm	group	
Ras	Cytoplasm	group	
RHOA	Cytoplasm	enzyme	387
SHP2	Cytoplasm	phosphatase	5781
SIP1(ZEB2)	Nucleus	transcription regulator	9839
SMAD2/3	Cytoplasm	group	
SMAD2/3-SMAD4	Nucleus	complex	
SMAD4	Nucleus	transcription regulator	4089
SMURF1	Cytoplasm	enzyme	57154
SNAIL-1	Nucleus	transcription regulator	6615
SNAIL-2(SLUG)	Nucleus	transcription regulator	6591
SOS	Cytoplasm	group	
STAT3	Nucleus	transcription regulator	6774
TACE	Plasma Membrane	peptidase	6868
TCF/LEF	Nucleus	group	
TGFβRI	Plasma Membrane	kinase	7046
TGFβRII	Plasma Membrane	kinase	7048
TGF-β	Extracellular Space	group	
TWIST	Other	group	
WNT	Extracellular Space	group	
ZEB	Other	group	
ZEB1	Nucleus	transcription regulator	6935

5- Genes of STAT3 Pathway

Symbol	Location	Family	Gene ID for Human
BCL2	Cytoplasm	transporter	596
CDC25A	Nucleus	phosphatase	993
CIS	Cytoplasm	other	1154
CytokineReceptor	Plasma Membrane	group	
Cytokines	Extracellular Space	group	
ERK1/2	Cytoplasm	group	
GF	Extracellular Space	group	
GFReceptor	Plasma Membrane	group	
GRIM-19	Cytoplasm	enzyme	51079
GTP	Other	chemical - endogenous mammalian	
JAK2	Cytoplasm	kinase	3717
JNK	Cytoplasm	group	
MKK1/2	Cytoplasm	group	
MKKs	Other	group	
MLKs	Other	group	
MYC	Nucleus	transcription regulator	4609
p21WAF1	Nucleus	kinase	1026
p38	Cytoplasm	group	
PIAS3	Nucleus	transcription regulator	10401
PIM1	Cytoplasm	kinase	5292
PTP	Cytoplasm	phosphatase	5771
RAC1	Plasma Membrane	enzyme	5879
RAF1	Cytoplasm	kinase	5894
RAS	Cytoplasm	group	
SHP1	Cytoplasm	phosphatase	5777
SOCS	Cytoplasm	group	
SRC	Cytoplasm	kinase	6714
STAT3	Nucleus	transcription regulator	6774
STAT3 dimer	Nucleus	complex	
Tumor Metastasis	Other	disease	
TYK2	Plasma Membrane	kinase	7297

6- Genes of Adipogenesis Pathway

Symbol	Location	Family	Gene ID for Human
βcatenin	Nucleus	transcription regulator	1499
AGPAT2	Cytoplasm	enzyme	10555
AKT	Cytoplasm	kinase	207
ATG5	Cytoplasm	other	9474
ATG7	Cytoplasm	enzyme	10533
BMAL1/CLOCK	Nucleus	complex	
BMP2/4/7	Extracellular Space	group	
BMPR	Plasma Membrane	complex	
BSCL2	Cytoplasm	other	26580
C/EBPα	Nucleus	transcription regulator	1050
C/EBPβ	Nucleus	transcription regulator	1051
C/EBPδ	Nucleus	transcription regulator	1052
CDK5	Nucleus	kinase	1020
CDK7	Nucleus	kinase	1022
CHOP-10	Nucleus	transcription regulator	1649
COUP-TFII	Nucleus	ligand-dependent nuclear receptor	7026
CTBP1/2	Nucleus	group	
DGKδ	Cytoplasm	kinase	8527
EBF1	Nucleus	transcription regulator	1879
EGR2	Nucleus	transcription regulator	1959
ETO	Nucleus	transcription regulator	862
EZH2	Nucleus	transcription regulator	2146
FABP4	Cytoplasm	transporter	2167
FBXW7	Nucleus	enzyme	55294
FGF-1	Extracellular Space	growth factor	2246
FGF-2	Extracellular Space	growth factor	2247
FGFR	Plasma Membrane	group	
FOXC2	Nucleus	transcription regulator	2303
FOXO1	Nucleus	transcription regulator	2308
FZD	Plasma Membrane	group	
GLUT4	Plasma Membrane	transporter	6517
HAT	Nucleus	complex	
HDAC	Other	complex	
HIF1A	Nucleus	transcription regulator	3091
IP6K	Nucleus	phosphatase	9677
KLF3	Nucleus	transcription regulator	51274
KLF5	Nucleus	transcription regulator	688
LEP	Extracellular Space	growth factor	3952
lipidsynthesis	Other	function	

LPIN1	Nucleus	phosphatase	23175
LPL	Cytoplasm	enzyme	4023
LSD1	Nucleus	enzyme	23028
miR-31	Cytoplasm	microRNA	407035
miR-326	Cytoplasm	microRNA	442900
MiR-448	Cytoplasm	microRNA	554212
miR155	Cytoplasm	microRNA	406947
MLL4	Nucleus	transcription regulator	9757
MNAT1	Nucleus	other	4331
NFATC4	Nucleus	transcription regulator	4776
NOC	Nucleus	transcription regulator	25819
p53	Nucleus	transcription regulator	7157
PER2	Nucleus	transcription regulator	8864
PLIN1	Cytoplasm	other	5346
PPARγ	Nucleus	ligand-dependent nuclear receptor	5468
PREF1	Extracellular Space	other	8788
PTIPβ	Nucleus	other	22976
RB	Nucleus	transcription regulator	5925
RBP1	Extracellular Space	transporter	5947
Rev-ERBα	Nucleus	ligand-dependent nuclear receptor	9975
S6K1	Other	group	
SENP2	Nucleus	peptidase	59343
SETDB1	Nucleus	enzyme	9869
SIRT1	Nucleus	transcription regulator	23411
SIRT2	Nucleus	transcription regulator	22933
SMAD1/5/8	Cytoplasm	group	
SMAD3	Nucleus	transcription regulator	4088
SOX9	Nucleus	transcription regulator	6662
SREBP1	Nucleus	transcription regulator	6720
STAT5	Nucleus	transcription regulator	6777
TCF7	Nucleus	transcription regulator	6932
TFIIH	Nucleus	complex	
TGFβ	Extracellular Space	growth factor	7040
TNFα	Extracellular Space	cytokine	7124
TNFR1	Plasma Membrane	transmembrane receptor	7132
TXNIP	Cytoplasm	other	10628
WNT10b	Extracellular Space	other	7480
WNT5a	Extracellular Space	cytokine	7474
XBP1	Nucleus	transcription regulator	7494
ZFP423	Nucleus	transcription regulator	23090

7- Genes of Glucocorticoid Receptor Signaling Pathway

Symbol	Location	Family	Gene ID for Human
14-3-3η	Cytoplasm	transcription regulator	7533
A2M	Extracellular Space	transporter	2
ADRB2	Plasma Membrane	G-protein coupled receptor	154
AGT	Extracellular Space	growth factor	183
AKT	Cytoplasm	group	
Aldosterone	Other	chemical - endogenous mammalian	
Aldosterone-MR	Cytoplasm	complex	
AMPK	Cytoplasm	complex	
ANF	Extracellular Space	other	4878
Ap-1	Nucleus	complex	
AR	Nucleus	ligand-dependent nuclear receptor	367
BAG1	Cytoplasm	other	573
BCL-2	Cytoplasm	transporter	596
BCL-XL	Cytoplasm	other	598
BGLAP	Extracellular Space	other	632
c-Fos	Nucleus	transcription regulator	2353
c-Jun	Nucleus	transcription regulator	3725
c-Raf	Cytoplasm	kinase	5894
Ca2+	Other	chemical - endogenous mammalian	
Calcineurin	Cytoplasm	complex	
CBP	Nucleus	transcription regulator	1387
CC10	Extracellular Space	cytokine	7356
CCL11	Extracellular Space	cytokine	6356
CCL13	Extracellular Space	cytokine	6357
CCL2	Extracellular Space	cytokine	6347
CCL3	Extracellular Space	cytokine	6348
CCL5	Extracellular Space	cytokine	6352
CD163	Plasma Membrane	transmembrane receptor	9332
CEBPα	Nucleus	transcription regulator	1050
CEBPβ	Nucleus	transcription regulator	1051
COX2	Cytoplasm	enzyme	5743
CREB	Nucleus	transcription regulator	1385
CSF2	Extracellular Space	cytokine	1437
CSN2	Extracellular Space	kinase	1447
CXCL2	Extracellular Space	cytokine	2921
DRIP150	Nucleus	transcription regulator	9282
DRIP205	Nucleus	transcription regulator	5469
Dynein	Cytoplasm	complex	
Elk-1	Nucleus	transcription regulator	2002
ER	Nucleus	ligand-dependent nuclear receptor	2099
ERK1/2	Cytoplasm	group	
Estrogen-ER	Nucleus	complex	

FCGR1	Plasma Membrane	transmembrane receptor	2209
FGG	Extracellular Space	other	2266
FKBP51	Nucleus	enzyme	2289
FKBP52	Nucleus	enzyme	2288
FOXO3A	Nucleus	transcription regulator	2309
GC-GCR dimer	Nucleus	complex	
GILZ	Nucleus	transcription regulator	1831
GR	Nucleus	ligand-dependent nuclear receptor	2908
GRB2	Cytoplasm	kinase	2885
HMG-1	Nucleus	transcription regulator	3146
HSP70	Cytoplasm	group	
HSP90	Cytoplasm	group	
IκB	Cytoplasm	group	
IκBα	Cytoplasm	transcription regulator	4792
ICAM1	Plasma Membrane	transmembrane receptor	3383
IFN-γ	Extracellular Space	cytokine	3458
Ikb-Rela-Nfkb1-Pkac	Cytoplasm	complex	
IKK	Cytoplasm	complex	
IL-1β	Extracellular Space	cytokine	3553
Il-10	Extracellular Space	cytokine	3586
IL-13	Extracellular Space	cytokine	3596
IL-2	Extracellular Space	cytokine	3558
IL-3	Extracellular Space	cytokine	3562
IL-4	Extracellular Space	cytokine	3565
IL-5	Extracellular Space	cytokine	3567
IL-6	Extracellular Space	cytokine	3569
IL-8	Extracellular Space	cytokine	3576
IL1R2	Plasma Membrane	transmembrane receptor	7850
IL1RA	Extracellular Space	cytokine	3557
iNOS	Cytoplasm	enzyme	4843
JAK1	Cytoplasm	kinase	3716
JAK2	Cytoplasm	kinase	3717
JAK3	Cytoplasm	kinase	3718
JNK	Cytoplasm	group	
Keratin	Nucleus	group	
Lipocortin	Plasma Membrane	enzyme	301
MEK1/2	Cytoplasm	group	
MEKK1	Cytoplasm	kinase	4214
MKK4/7	Cytoplasm	group	
MKP-1	Nucleus	phosphatase	1843
MMP1	Extracellular Space	peptidase	4312
MR	Nucleus	ligand-dependent nuclear receptor	4306
NCOA2	Nucleus	transcription regulator	10499
NCOA3	Nucleus	transcription regulator	8202
NCOR	Nucleus	group	
NF-κB1 p50	Nucleus	transcription regulator	4790

NFAT	Cytoplasm	group	
NfkB-RelA	Nucleus	complex	
NIK	Cytoplasm	kinase	9020
OCT1/2	Nucleus	group	
p21Cip1	Nucleus	kinase	1026
p300	Nucleus	transcription regulator	2033
p38 MAPK	Cytoplasm	group	
p57Kip2	Nucleus	other	1028
p65/RelA	Nucleus	transcription regulator	5970
PAI-1	Extracellular Space	other	5054
PBX	Nucleus	transcription regulator	5087
PCAF	Nucleus	transcription regulator	8850
PEPCK	Nucleus	group	
PGR	Nucleus	ligand-dependent nuclear receptor	5241
PI3K	Cytoplasm	complex	
PKAc	Cytoplasm	group	
POMC	Extracellular Space	other	5443
PRL	Extracellular Space	cytokine	5617
Rac1	Plasma Membrane	enzyme	5879
Ras	Cytoplasm	group	
RIP140	Nucleus	transcription regulator	8204
RNAPol II	Nucleus	complex	
RNA pol II-Transcription factors	Nucleus	complex	
SELE	Plasma Membrane	transmembrane receptor	6401
SGK1	Cytoplasm	kinase	6446
SHC	Cytoplasm	other	6464
SLPI	Cytoplasm	other	6590
SMAD2/3	Cytoplasm	group	
Smad2/3-Smad4	Nucleus	complex	
SMAD4	Nucleus	transcription regulator	4089
SMILE	Nucleus	transcription regulator	58487
SOS	Cytoplasm	group	
SRA1	Nucleus	transcription regulator	10011
SRC-1	Nucleus	transcription regulator	8648
STAT1	Nucleus	transcription regulator	6772
Stat1 dimer	Nucleus	complex	
STAT3	Nucleus	transcription regulator	6774
Stat3 dimer	Nucleus	complex	
STAT5	Cytoplasm	group	
Stat5 dimer	Nucleus	complex	
SUMO1	Nucleus	enzyme	7341
SWI/SNF	Nucleus	complex	
TAB1	Cytoplasm	enzyme	10454
TAF	Nucleus	complex	
TAK1	Cytoplasm	kinase	6885

TAT	Cytoplasm	enzyme	6898
TBP	Nucleus	transcription regulator	6908
TCRα	Plasma Membrane	transmembrane receptor	6955
TCRβ	Plasma Membrane	transmembrane receptor	6957
TEBP	Cytoplasm	enzyme	10728
TFIIA	Nucleus	complex	
TFIIB	Nucleus	transcription regulator	2959
TFIIE	Nucleus	complex	
TFIIF	Nucleus	complex	
TFIIH	Nucleus	complex	
Tgf beta receptor	Plasma Membrane	complex	
TGF-β	Extracellular Space	group	
TGFBR1	Plasma Membrane	kinase	7046
TGFBR2	Plasma Membrane	kinase	7048
TNFα	Extracellular Space	cytokine	7124
TRAF2	Cytoplasm	enzyme	7186
TRAF6	Cytoplasm	enzyme	7189
TSG101	Cytoplasm	transcription regulator	7251
UBC9	Nucleus	enzyme	7329
uPA	Extracellular Space	peptidase	5328
VCAM1	Plasma Membrane	transmembrane receptor	7412
VIPR1	Plasma Membrane	G-protein coupled receptor	7433

8- **Genes of Beta Cell Receptor Signaling Pathway**

Symbol	Location	Family	Gene ID for Human
ABL1	Nucleus	kinase	25
AKT	Cytoplasm	group	
APBB1IP	Cytoplasm	other	54518
ATF-2	Nucleus	transcription regulator	1386
BAD	Cytoplasm	other	572
BAM32	Cytoplasm	other	27071
BCAP	Cytoplasm	kinase	118788
BCL-10	Cytoplasm	transcription regulator	8915
BCL-6	Nucleus	transcription regulator	604
BCL-XL	Cytoplasm	other	598
Bcl10-Card10-Malt1	Cytoplasm	complex	
BCR	Plasma Membrane	complex	
BFL-1	Cytoplasm	other	597
BLNK	Cytoplasm	other	29760
BTK	Cytoplasm	kinase	695
c-Jun	Nucleus	transcription regulator	3725
c-RAF	Cytoplasm	kinase	5894
CaM	Cytoplasm	group	
CaMKII	Cytoplasm	complex	
CARD11	Cytoplasm	other	29775
CD19	Plasma Membrane	transmembrane receptor	930
CD22	Plasma Membrane	transmembrane receptor	933
CD45	Plasma Membrane	phosphatase	5788
CD79A	Plasma Membrane	transmembrane receptor	973
CD79B	Plasma Membrane	transmembrane receptor	974
Cofilin	Cytoplasm	group	
CREB	Nucleus	group	
CSK	Cytoplasm	kinase	1445
EBF1	Nucleus	transcription regulator	1879
EBF1-FOXO1-TCF3	Nucleus	complex	
EGR-1	Nucleus	transcription regulator	1958
ELK-1	Nucleus	transcription regulator	2002
ERK1/2	Cytoplasm	group	
ETS-1	Nucleus	transcription regulator	2113
FAK	Cytoplasm	group	
FCγRII	Plasma Membrane	group	
FOXO1	Nucleus	transcription regulator	2308
GAB1/2	Cytoplasm	group	
GRB2	Cytoplasm	kinase	2885
GSK3	Cytoplasm	group	
IκB	Cytoplasm	group	
IGG	Extracellular Space	complex	

IkB-NfkB	Cytoplasm	complex	
IKK	Cytoplasm	complex	
JNK1/2	Cytoplasm	group	
LYN	Cytoplasm	kinase	4067
MALT1	Cytoplasm	peptidase	10892
MEF2C	Nucleus	transcription regulator	4208
MEK1/2	Cytoplasm	group	
MEKK	Cytoplasm	group	
MKK3/4/6	Cytoplasm	group	
MKK4/7	Cytoplasm	group	
mTOR	Nucleus	kinase	2475
NF-κB	Nucleus	complex	
NFAT	Cytoplasm	complex	
OCT-2	Nucleus	transcription regulator	5452
p38 MAPK	Cytoplasm	group	
p70S6K	Cytoplasm	group	
PAG	Plasma Membrane	other	55824
PAX5	Nucleus	transcription regulator	5079
PDK1	Cytoplasm	kinase	5170
PI3K	Cytoplasm	complex	
PKC(β,θ)	Cytoplasm	group	
PLCγ2	Cytoplasm	enzyme	5336
PP2B	Cytoplasm	complex	
PTEN	Cytoplasm	phosphatase	5728
PTK2B	Cytoplasm	kinase	2185
RAC/CDC42	Cytoplasm	group	
RAP	Cytoplasm	group	
RAS	Cytoplasm	group	
RASSF5	Plasma Membrane	other	83593
SHC	Cytoplasm	other	6464
SHIP	Cytoplasm	group	
SHP1	Cytoplasm	phosphatase	5777
SHP2	Cytoplasm	phosphatase	5781
SOS	Cytoplasm	group	
Syk	Cytoplasm	kinase	6850
TCF3	Nucleus	transcription regulator	6929
VAV	Cytoplasm	group	

9- Genes of PTEN (Phosphatase and tensin homolog) Signaling Pathway

Symbol	Location	Family	Gene ID for Human
14-3-3η	Cytoplasm	transcription regulator	7533
AKT	Cytoplasm	group	
BAD	Cytoplasm	other	572
BCL-2	Cytoplasm	transporter	596
BCL-XL	Cytoplasm	other	598
BIM	Cytoplasm	other	10018
c-Raf	Cytoplasm	kinase	5894
CAS	Plasma Membrane	enzyme	9564
CASP3	Cytoplasm	peptidase	836
CASP9	Cytoplasm	peptidase	842
CBL	Nucleus	transcription regulator	867
CDC42	Cytoplasm	enzyme	998
CK2	Cytoplasm	complex	
CyclinD1	Nucleus	transcription regulator	595
ERK1/2	Cytoplasm	group	
FAK	Cytoplasm	kinase	5747
FASL	Extracellular Space	cytokine	356
FOXO	Cytoplasm	group	
GFR	Plasma Membrane	group	
GRB2	Cytoplasm	kinase	2885
GSK3	Cytoplasm	group	
IKK	Cytoplasm	complex	
ILK	Plasma Membrane	kinase	3611
Integrin	Plasma Membrane	complex	
MAGI	Cytoplasm	group	
Magi-Pten	Cytoplasm	complex	
MAST2	Cytoplasm	kinase	23139
MEK1/2	Cytoplasm	group	
MSP58	Nucleus	other	10445
NF-κB	Nucleus	complex	
p21CIP1	Nucleus	kinase	1026
p27KIP1	Nucleus	kinase	1027
p70 S6K	Cytoplasm	group	
PDK1	Cytoplasm	kinase	5170
PI3K p101	Cytoplasm	kinase	23533
PI3K p110γ	Cytoplasm	kinase	5294
PI3K p110	Cytoplasm	group	
PI3K p85	Cytoplasm	group	
PKCζ	Cytoplasm	kinase	5590
PREX2	Cytoplasm	other	80243
PTEN	Cytoplasm	phosphatase	5728
RAC	Cytoplasm	group	

Ras	Cytoplasm	group	
SHC	Cytoplasm	other	6464
SHIP	Cytoplasm	group	
SIPL1	Plasma Membrane	other	81858
SOS	Cytoplasm	group	
